# Use of Vancomycin Mixed Bone Graft and Vancomycin Mixed Saline Wash Before Wound Closure Reduces the Rate of Infection in Lumbar Spine Fusion Surgery

**DOI:** 10.7759/cureus.17275

**Published:** 2021-08-18

**Authors:** Bikram Kar, Nagaraju Venishetty, Sandeep Kumar Yadav, Harshal Sakale

**Affiliations:** 1 Orthopaedics, All India Institute of Medical Sciences, Raipur, IND

**Keywords:** lumbar spine fusion, intraoperative vancomycin saline wash, postoperative infection, spinal surgery infection prophylaxis, surgical site vancomycin prophylaxis

## Abstract

This study aims to evaluate whether surgical site vancomycin mixed in bone grafts and local vancomycin mixed in normal saline wash before wound closure decrease the infection rates in patients undergoing lumbar spinal instrumentation and posterolateral fusion. A retrospective study was performed on cases between 2017 and 2019, who underwent lumbar spine instrumentation and posterolateral fusion for lumbar canal stenosis or listhesis. The routine prophylactic procedures were performed in all patients as per institutional protocol. Patients' records were analysed and categorised into two groups, the vancomycin group (VG), where vancomycin mixed in bone graft and normal saline wash was used at the surgical site, and the control group (CG), where vancomycin was not used. The study included 63 patients, 31 in VG and 32 in CG. There is no statistical difference in age, sex, and diabetes mellitus in both groups. A total of seven cases were infected, six in CG (6/32) and one in VG (1/31). Out of six infections in CG, three patients had diabetes and four infected cases underwent surgery for debridement. In VG, the only single case got infected and treated with intravenous antibiotics. We found that the use of vancomycin added to the bone graft and normal saline in posterior lumbar spinal instrumentation and posterolateral fusion is associated with significantly lower rates of infection (p value=0.049).

## Introduction

Infection after spinal surgery with instrumentation is a devastating complication that increases patient morbidity, mortality, and treatment costs as multiple surgeries and specific antibiotics are used [1]. Many studies have shown well-defined risk factors for infection after spinal surgery. Elderly age, obesity, poor nutrition, prolonged surgical time, increased blood loss, smoking, implants, and revision surgery are few among them [2]. The rates of these postoperative infections after spinal surgeries have to be documented accurately as it helps to improve the quality of treatment, proper patient counselling, and surgical decision making. Deep infection after spinal surgery appears to be a more accurate parameter for research documentation than superficial infections, whose definitions are complicated by including minor temporary discharge, suture reactions, or wound erythema [3,4]. Superficial infections are those limited to layers above the fascial layer, in the skin and subcutaneous tissues. Deep infections are those that extend below the fascia (lumbodorsal, platysma, ligamentum nuchae, or anterior abdominal fascia, depending on surgical site). Most deep infections usually require surgical debridement as a standard treatment. In spinal surgeries, decompression or fusion, without instrumentation, postoperative surgical site infection rates range from 0.7% to 2.3% and instrumentation in spinal surgeries increases this rate from 0.3% to 20% [2,5,6]. Delayed infection after posterior spinal instrumentation ranges between 0.2% and 6.7% [7]. The most common organism causing postoperative spinal infections is *Staphylococcus aureus*, and other organisms include *Staphylococcus epidermidis*, *Enterococcus faecalis*, *Pseudomonas* spp., *Enterobacter cloacae*, and *Proteus mirabilis* [8]. Recently, in a study by Koutsoumbelis et al., 34% of surgical site infections (SSIs) demonstrated positive cultures for Methicillin-resistant *Staphylococcus aureus* (MRSA), indicating an increasing prevalence of this organism [9]. Postoperative surgical site infections after instrumented spinal surgery are a challenge to treat [7]. To decrease the rates of these infections, surgeons should minimize perioperative risk factors of infection. Literature shows that aseptic surgical techniques and intravenous antibiotic prophylaxis have effectively prevented postoperative infections [10-12]. Of late, surgeons are practising additional decontamination of the wound before closure with antibiotics, like vancomycin, directly into the wound or antiseptic irrigation with povidone-iodine and hydrogen peroxide [13-16]. Molinari et al., in their study, concluded that the use of vancomycin powder placed in the wound before wound closure had shown a low rate of deep spinal wound infection in both instrumented and uninstrumented cases [2]. In another study, both the use of antibiotics and antiseptic intrawound prophylactics reduced deep surgical site infections in instrumented spine surgery significantly by three to seven times, without any adverse reactions [17].

We hypothesize that the use of local vancomycin in normal saline wash and in bone graft decreases the rate of postoperative infections. The present study evaluates the efficacy of surgical site vancomycin mixed bone graft and local vancomycin mixed saline wash before wound closure in decreasing infection rates in patients undergoing lumbar instrumented spine fusion.

## Materials and methods

This study is a retrospective study conducted at a tertiary healthcare facility. The study included all the consecutive patients who underwent posterior lumbar spinal instrumentation and posterolateral fusion with local bone graft, with indications including lumbar canal stenosis or listhesis, between January 2017 and December 2019. Recorded data of patients were collected and analysed systemically. We excluded the patients in whom surgeries were performed through an anterior approach, surgeries were performed without fusion, cervical and thoracic surgeries were done, and tumours and infections were found. All the surgeries included in the study are performed by the first author. The instruments used are from the same company and are subject to the same sterilization method as per institutional protocols. All patients were given surgical prophylaxis as per institutional protocol. The institutional protocol includes a 1.5gm cefuroxime intravenous injection within one hour before skin incision. After anaesthesia, the patient is placed in a prone position over bolsters. Level of spinal surgery is identified and marked. Surgical area scrubbed with 7.5% betadine solution and painted with 10% betadine solution. Sterile surgical drapes were applied over the surgical area. Surgical site painted with 2% chlorhexidine solution. Local diluted adrenaline, one ampoule (1mg) in 100ml normal saline, was given around the surgical site to decrease perioperative bleeding and for a bloodless surgical field. Posterior lumbar spinal instrumentation and posterolateral fusion were done with a local bone graft (autograft obtained from laminectomy for decompression during surgery/posterior iliac crest). Postsurgery wound wash was performed with normal saline and betadine routinely. Drains were placed subfascial and left in place for 48 hours. Postoperative antibiotic prophylaxis was performed with injection cefuroxime 1.5gm and amikacin 500mg intravenously twice a day for two days, followed by oral cefuroxime 500mg twice a day for five days, which is routinely followed at our centre. Postoperative dressings were done on days two and five. Further dressings are done based on soakage and discharge from the surgical site and followed up to 12 months post surgery.

Patients were categorised into two groups based on the use of vancomycin with bone graft and saline wash at the time of wound closure: vancomycin group (VG), where vancomycin mixed in bone graft and normal saline wash was used at the surgical site, and control group (CG), where vancomycin was not used. Vancomycin is chosen as it has better diffusion characteristics than other antibiotics [18]. Powdered vancomycin with bone graft may also act as a scaffold for bone formation and fusion. Vancomycin mixed saline wash was given (1gm in 100ml). The antibiotic, 1gm of vancomycin, was mixed with bone graft (autograft obtained from laminectomy for decompression during surgery/posterior iliac crest) and a small amount of the patient’s blood (from surgical site) to promote adhesion of the antibiotics to bone graft, 5-15 minutes before placing them in the posterolateral region of the lumbar spine. No routine normal saline wash is given after vancomycin wash.

The study patients' medical and surgical records searched for evidence of postoperative infection and treatment received for the same. All the patients in the study with postoperative infection were treated either with prolonged intravenous antibiotics or with surgical debridement, the record of which further helped in easy analysis of data in finding infection numbers. For statistical analysis, the data were entered into a Microsoft Excel spreadsheet, and analysis was done using SPSS Statistics software. A Chi-square test is used, and a p-value <0.05 was considered statistically significant.

## Results

**Figure 1:**
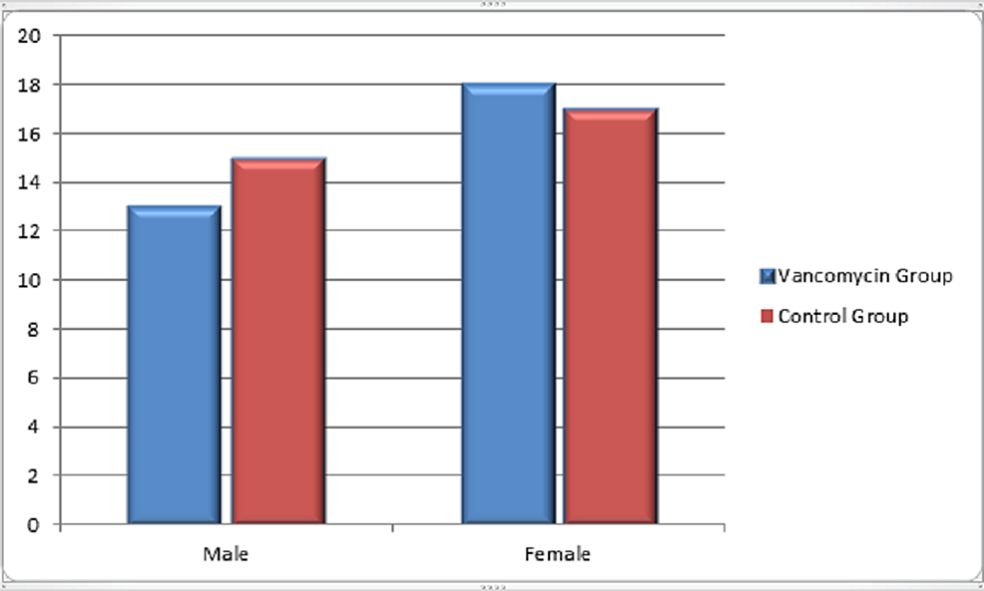
Sex distribution in vancomycin and control groups

**Figure 2:**
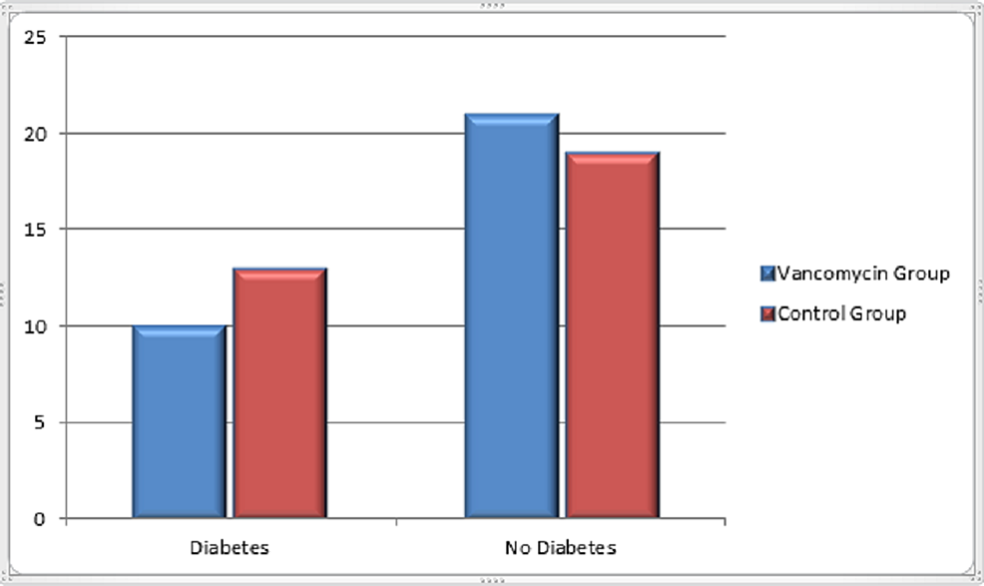
Diabetes in vancomycin and control groups

**Figure 3:**
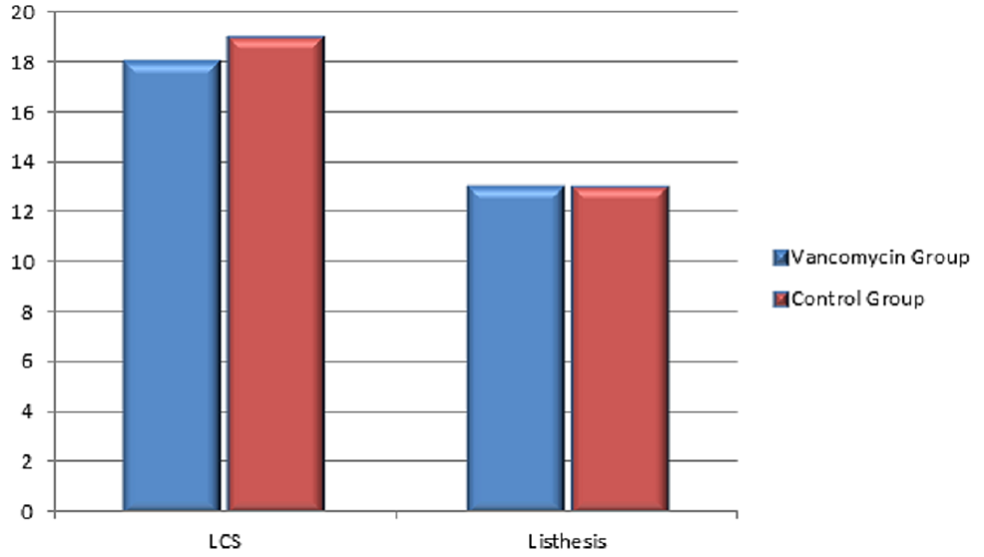
Diagnosis in vancomycin and control groups LCS: lumbar canal stenosis.

**Figure 4:**
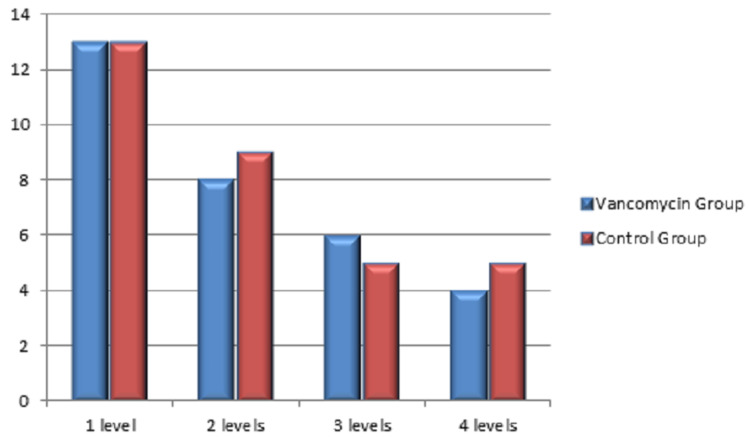
Number of vertebral levels in surgery in vancomycin and control groups

In this retrospective study, a total of 63 patients were included. Among them, 31 patients received vancomycin mixed bone graft and vancomycin mixed normal saline wash (VG) during surgery, and 32 patients received bone graft and washed without antibiotic (CG). The statistical analysis showed a mean age of 49.31 years. As shown in Table 1, there was no statistical difference between the groups in variables concerning sex (VG: 58.1% females, 41.9% males in comparison with 53.1% females and 46.9% males in CG, p value=0.693), diabetes (32.3% in VG and 40.6% in CG with p value=0.490), diagnosis (VG: 58.1% lumbar canal stenosis and 41.9% listhesis against 59.4% and 40.6%, respectively, in CG with p value=0.9159), and also the number of spinal levels with p value of 0.97 which is insignificant. Figure 1 shows the sex distribution among the groups. Figure 2 shows the distribution of diabetic patients in both groups. Distribution of diagnosis in CG and VG is shown in Figure 3. Also the distribution of number of vertebral levels in CG and VG is shown in Figure 4.

**Table 1:** Demographic data and comparison of variables between vancomycin group and control group

	Total (N=63)	Control Group (N=32)	Vancomycin Group (N=31)	P Value
Age (Average)	49.31 years	48.03 years	50.58 years	
Males	28	15	13	0.693
Females	35	17	18	0.693
Diabetes	23	13	10	0.4904
Lumbar Canal Stenosis	37	19	18	0.9159
Listhesis	26	13	13	0.9159
Postoperative Infections	7	6	1	0.0499

**Figure 5:**
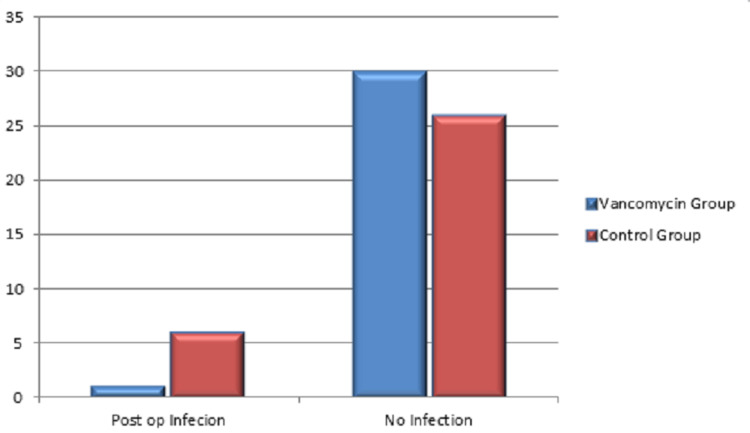
Postoperative infection in vancomycin and control groups Post op: postoperative.

Postoperative infection was seen in six cases in CG (6/32), i.e., 18.75%, and one in the vancomycin group got infected (1/31), i.e., 3.2%, with a p value of 0.049 which is significant. Figure 5 shows the distribution of postoperative infections in VG and CG. As per data records, postoperative wound infection was suspected if there is persistent discharge at the surgical site with local signs of inflammation, clinical febrile (>99°F), and chills and supported by laboratory studies such as complete blood picture (white blood cells more than 11,000 per microlitre), erythrocyte sedimentation rate (>25mm/hr, raising after the fourth postoperative day), and C-reactive protein (>5mg/ml after an initial decline for two days). Diagnosis confirmed with positive cultures with antibiotic sensitivity test. According to the culture report, infected cases were treated with sensitive antibiotics and monitored with clinical and laboratory parameters for improvement. Patients deteriorating and not improving even with parenteral antibiotics underwent surgical debridement. 

Among six cases infected in the control group, three were diabetic. Culture reports of infected patients showed three organisms, *Klebsiella pneumoniae* (one case), *Escherichia coli* (one case), and *S. aureus* (three cases). In two of six infected cases (superficial infections), infection subsided, and patients recovered fully with only proper antibiotic treatment, both injectable and oral, as per culture and sensitivity. The remaining four cases (deep infections) required a second surgery, in the form of debridement and wash. Only one case (superficial infection) got infected among the vancomycin group, with *E. coli* treated with intravenous and oral antibiotics to the complete cure. No other complications were observed in either group.

## Discussion

Surgical site infections are a significant concern in spinal instrumentation and fusion surgeries as they cause increased morbidity, mortality, and financial burden for the patient. Current literature gives an incidence between 2.6 and 3.8 for infections after posterior spinal instrumentation [7,19]. Risk factors for infection after spinal surgery include elderly age, obesity, poor nutrition, prolonged surgical time, increased blood loss, smoking, implants, and revision surgery [2]. *S. aureus* is the most common organism isolated with an increasing incidence of MRSA [8,9]. Many studies were done on methods to decrease these infections. The North American Spine Society has published evidence-based guidelines that all patients undergoing spinal surgery should receive perioperative prophylactic antibiotics [20]. Chang et al. and Cheng et al. reported that irrigation of surgical site with dilutions of betadine showed promising results with no infection [21,22]. Recently, there are studies on local application of powdered antibiotics in the wound before closure to reduce infection rates [2]. Theoretically, the local application of powdered antibiotics gives high concentrations of antibiotics in the operative area for a prolonged period. There are many local antimicrobial strategies like usage of polymethyl methacrylate (PMMA), bone grafts, calcium phosphate, and calcium sulphate. PMMA, a nonbiodegradable cement, lose antibiotic elution function in few days. Being avascular, they are vulnerable to bacterial colonization. Other disadvantages are the need for a second surgery to remove them and a limited choice of antibiotics to use because of the heat produced during setting. Above problems would be overcome by locally delivering antibiotics through a biodegradable substance, with a suitable elution profile, like bone grafts [23]. Several approaches resulted in high initial antibiotic concentrations, essential for prophylaxis and even biofilm eradication. In comparison with cancellous bone, cortical bone is less accessible and resulted in lower antibiotic elution concentrations [24]. Special impregnation methods may be useful in modifying the antibiotic elution profile. Vancomycin was found to be the least osteotoxic antibiotic with a good elution profile. With powdered vancomycin (1%w/w), elution was well above the minimum inhibition concentration (MIC) for *S. aureus*, with a maximum of 499.7µg/ml [25]. In another study, cancellous grafts impregnated with liquid vancomycin (100mg/ml) reached initial concentrations up to 20,000µg/ml [24]. This is well above the toxic dose of vancomycin described in a study where 10,000µg/ml vancomycin caused osteoblastic cell death, while 1,000µg/ml had no effect [23]. This might be the reason for use of powdered vancomycin in most studies. Molinari et al. in their study concluded that the use of vancomycin powder placed in the wound before wound closure had shown a low rate of deep spinal wound infection in both instrumented and uninstrumented cases [2]. O'Neill et al. and Sweet et al. observed a decrease in surgical site infections in instrumented spinal surgery using local powdered vancomycin before wound closure [26,27]. In another study, both the use of antibiotics and antiseptic intrawound prophylactics reduced deep surgical site infections in instrumented spine surgery significantly by three to seven times, without any adverse reactions [17]. The limitations of many of these studies were the lack of a control group. The present study is a retrospective case-control study. The mean age in our study (50.76) is in support of the previous study [6]. Our study showed a significant decrease (p value=0.01) in postoperative surgical infections after posterior lumbar spinal instrumentation and posterolateral fusion using powdered vancomycin mixed with bone graft and normal saline for a wash before wound closure. These results are consistent with the above-mentioned studies. In this study, no systemic toxicity was observed in any of the VG patients, and the same was marked by Lemans et al. [17].

There are limitations to our study. This is a retrospective study with a small number of subjects and only 12-month follow-up. There is no blinding or randomization of subjects. Further risk factors for infection, like the length of surgical time, comorbidities, etc., are not discussed. We also did not include secondary outcomes of the surgery like nonunion, functional improvement, etc.

## Conclusions

Before wound closure, local use of powdered vancomycin to mix with bone graft and normal saline for wash could be a simple and effective way to prevent devastating complications of surgical site infections after posterior spine instrumentation and fusion surgery. Further studies in this regard with a more extensive study group, long-term follow-up, and prospective study with better randomisation methodology are required to confirm these results.
